# Ventilator-associated pneumonia involving *Aspergillus flavus* in a patient with coronavirus disease 2019 (COVID-19) from Argentina

**DOI:** 10.1016/j.mmcr.2020.07.001

**Published:** 2020-07-05

**Authors:** Norma B. Fernandez, Diego H. Caceres, Karlyn D. Beer, Célica Irrazabal, Ghilka Delgado, Luciana Farias, Tom M. Chiller, Paul E. Verweij, Daniel Stecher

**Affiliations:** a Sección Micología, División Infectología, Hospital de Clínicas “José de San Martin”, Universidad de Buenos Aires, Ciudad Autónoma de Buenos Aires, Argentina; b Mycotic Diseases Branch, Centers for Disease Control and Prevention, Atlanta, GA, USA; c Department of Medical Microbiology, Radboud University Medical Center and Center of Expertise in Mycology Radboudumc/CWZ, Nijmegen, the Netherlands; d División Terapia Intensiva, Hospital de Clínicas “José de San Martín”Ciudad Autónoma de Buenos Aires, Argentina

**Keywords:** Aspergillosis, COVID-19, Coronavirus, Ventilator-associated pneumonia, Intensive care unit

## Abstract

Coronavirus disease 2019 (COVID‐19), caused by the novel coronavirus SARS-CoV-2, emerged in Wuhan, China, in December 2019 and rapidly spread around the world. Invasive aspergillosis has been reported as a complication of severe influenza pneumonia among intensive care patients. Similarities between COVID-19 and influenza pneumonia, together with limited published case series, suggest that aspergillosis may be an important complication of COVID-19. This report describes a case of ventilator-associated pneumonia involving *Aspergillus flavus* in a patient with COVID-19 from Buenos Aires, Argentina.

## 1 Introduction

SARS-CoV-2 is the novel coronavirus that causes coronavirus disease 2019 (COVID‐19), which was first reported in December 2019 in Wuhan, China, and has rapidly spread worldwide [1]. On March 11^th^, 2020, the World Health Organization (WHO) declared a pandemic, and by June 30^th^, 2020, a total of 10,185,374 cases and 503,862 deaths were reported in 216 locations around the world [2]. In Argentina, on June 30^th^, 2020, the Argentinean Ministry of Health (AMOH) reported 62,268 cases and 1283 deaths [3].

Invasive pulmonary aspergillosis (IPA) is a known complication in patients with severe influenza pneumonia. Case series from the Netherlands and Belgium reported an incidence of 19% in patients with influenza pneumonia and acute respiratory distress syndrome (ARDS) hospitalized in the intensive care unit (ICU) [4], although these high incidences have not been confirmed in other studies [4]. There is concern that critically ill COVID-19 patients might also be at increased risk of pulmonary aspergillosis co-infection [5]. Here we describe a report of ventilator-associated pneumonia involving *Aspergillus flavus* in a patient with COVID-19 and ARDS in Argentina.

## 2 Case presentation

**Fig. 1:**
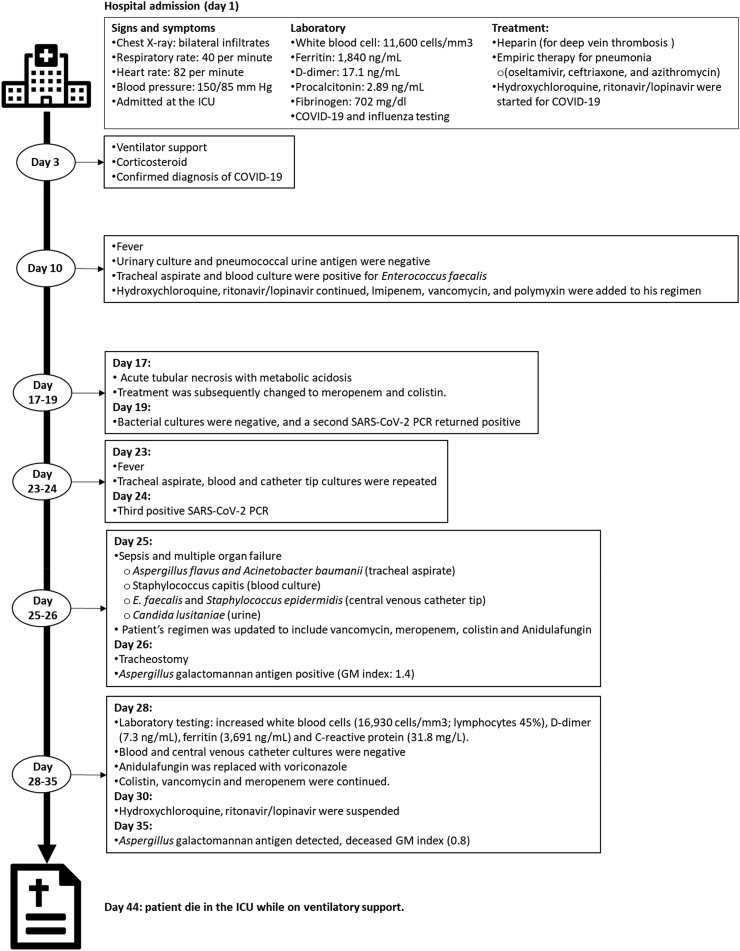
Case timeline: ventilator-associated pneumonia involving *Aspergillus flavus* in a patient with coronavirus disease 2019 (COVID-19) from Argentina.

In late April 2020, an 85-year-old man with a history of hypertension was admitted to the ICU with a history of six days of fever and dyspnea, at home. The patient had close household contact with two COVID-19 cases, and nasopharyngeal (NP) swab samples collected on the day of ICU admission tested positive for SARS-CoV-2 using molecular testing. On ICU admission (day 1) the patient presented with bilateral infiltrates (ground-glass opacities) on chest radiograph, respiratory rate of 40 breaths per minute, heart rate of 82 beats per minute, and a blood pressure 150/85 mm Hg. Laboratory tests showed a white blood cell count of 11,600 cells/mm^3^, with lymphopenia, normal serum creatinine (1.02 mg/dL), low albumin (2.8 g/dL), and normal lactate (1.4 mmol/L), as well as elevated ferritin (1840 ng/mL), D-dimer (17.1 ng/mL), procalcitonin (2.89 ng/mL), and fibrinogen (702 mg/dl). The patient also presented with deep vein thrombosis in the right subclavian vein and in the right internal jugular vein; anticoagulation was initiated with heparin due to the hypercoagulable state. Empirical treatment for pneumonia was started with oseltamivir, ceftriaxone, and azithromycin, and hydroxychloroquine, ritonavir/lopinavir were started for COVID-19, according to the Argentinean Ministry of Health (AMOH) national guidelines [6]. After 3 days of hospitalization, the patient required ventilator support (orotracheal intubation and mechanical ventilation) and was given a corticosteroid (dexamethasone, 20 mg/day). Influenza antigen testing yielded a negative result, prompting cessation of empirical influenza treatment (Fig. 1).

On day 10 of hospitalization, the patient developed a new fever (38 °C). Urinary culture and pneumococcal urine antigen test were negative; tracheal aspirate and blood culture were positive for *Enterococcus faecalis,* and imipenem and vancomycin were added to hydroxychloroquine and ritonavir/lopinavir. Two days later polymyxin was added, and blood cultures turned negative after seven days of treatment. On day 17, urinary analysis showed evidence of acute tubular necrosis with metabolic acidosis (low pH, PaCO_2_ and HCO^3^), and treatment was subsequently changed to meropenem and colistin. On day 19, tracheal aspirate, urine and blood culture remained negative for bacteria, and a second SARS-CoV-2 PCR test from a NP swab was positive. On day 23, the patient was again febrile, and urinary, tracheal aspirate, blood and catheter tip cultures were repeated; a third SARS-CoV-2 PCR test on day 24 from a NP swab was positive (Fig. 1).

**Fig. 2:**
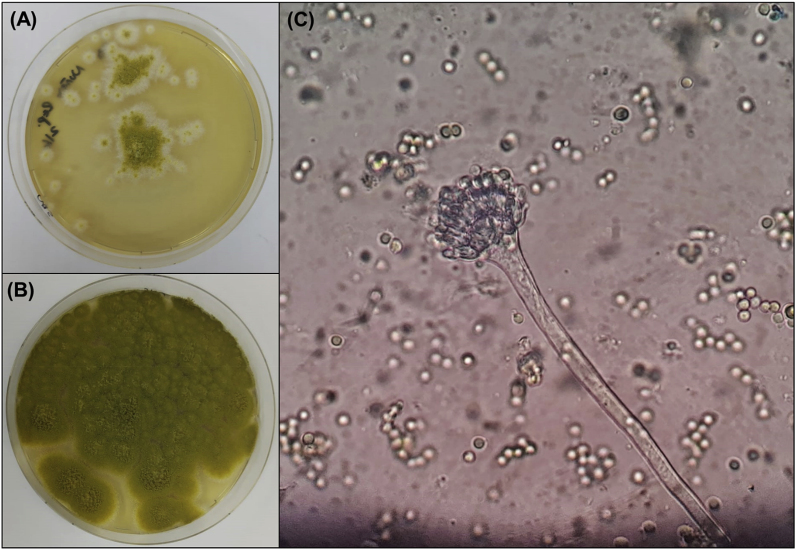
*Aspergillus flavus* isolate: (A and B) Macroscopic: rapid growth olive green colonies, with woolly texture, on Sabouraud agar incubated at 28 °C (A) and 37 °C (B). (C) Microscopic: hyaline septate hyphae, radiate biseriate conidial head with finely roughened conidiophore. (For interpretation of the references to colour in this figure legend, the reader is referred to the Web version of this article.)

**Fig. 3:**
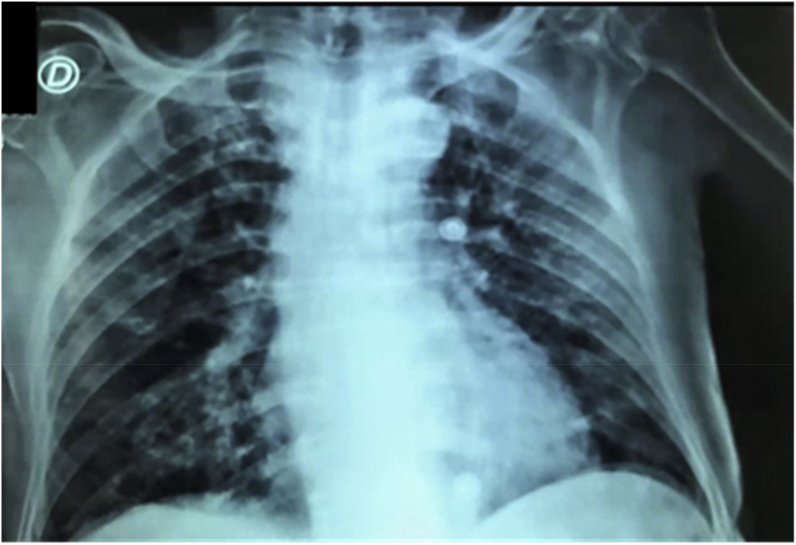
Chest X-ray evidenced bilateral heterogeneous infiltrates on day 26 of hospitalization: picture from the COVID-19 associated pulmonary aspergillosis (CAPA) described on this report.

On day 25 the patient developed multiple organ failure, including acute renal failure. *Aspergillus flavus,* identified by MALDI-TOF (Fig. 2), and *Acinetobacter baumanii* (>10^6^ CFU) were cultured from tracheal aspirate. *Staphylococcus capitis* was isolated from blood culture, and *E. faecalis* and *Staphylococcus epidermidis* were isolated from the central venous catheter tip culture. *Candida lusitaniae* was isolated from urine. The patient's antimicrobial regimen was updated to include vancomycin, meropenem, and colistin. Anidulafungin was added to control candidiasis, but azole therapy for the *A. flavus* finding was not started due to initial clinical suspicion of colonization versus infection. On day 26 a tracheostomy was placed as a result of prolonged ventilation, ARDS (PaO2/FiO2 ratio <200), and sepsis. Chest radiograph continued to show bilateral infiltrates (Fig. 3). *Aspergillus* galactomannan antigen (GM) was positive in serum (GM index: 1.4; Platelia *Aspergillus;* Bio-Rad, Marnes-La-Coquette, France) (Fig. 1).

On day 28, laboratory testing showed increased white blood cells (16,930 cells/mm^3^; lymphocytes 45%), D-dimer (7.3 ng/mL), ferritin (3691 ng/mL) and C-reactive protein (31.8 mg/L). Blood and central venous catheter cultures were negative, and anidulafungin was replaced with voriconazole (400 mg first day, and then 300 mg daily) after the GM test returned positive. Colistin, vancomycin, and meropenem were continued. On day 30, hydroxychloroquine, ritonavir/lopinavir were suspended (following recommendation of AMOH), and on day 35, serum GM index decreased to 0.8. On day 44, the patient died in the ICU while on ventilatory support (Fig. 1).

## 3 Discussion

COVID-19 associated pulmonary aspergillosis (CAPA) has been recently reported in European countries [[7], [8], [9], [10], [11], [12], [13]], including four case series covering 27 patients from France (n = 9), Belgium (n = 7), The Netherlands (n = 6), and Germany (n = 5) [[7], [8], [9], [10]]. Our case has similar clinical features compared with those previously reported. Among these four case series, all 27 patients were admitted to the ICU and developed ARDS. Most were male, >50 years old, and 25 (89%) of 27 had potential risk factors for severe COVID-19. Three (60%) of five patients from the German case series, seven (78%) of nine from the French case series, and three (43%) of seven patients from the Belgium case series had hypertension. All five patients from Germany had acute kidney injury, and three (33%) of nine patients from France and two (29%) of seven from Belgium received renal replacement therapy [[7], [8], [9], [10]]. Abnormal chest radiologic findings were reported in all of the patients from Germany (n = 5) and France (n = 9) [7,8]. Twelve (44%) of 27 patients received corticosteroids during their ICU stay, and 19 (70%) of 27 were treated with mold-active antifungal medication, eight (42%) of these 19 patients received corticosteroid simultaneous with antifungal treatment. Fifteen (55%) of the 27 case series patients died. Our patient received corticosteroids for 22 days before initial *A. flavus* culture and was treated with voriconazole starting 3 days after the initial fungal culture. Unlike influenza-associated pulmonary aspergillosis (IAPA), which commonly occurs early after ICU admission [4], our patient developed CAPA 25 days after ICU admission. COVID-19 associated pulmonary aspergillosis case series from Belgium and The Netherlands, showed variable time on development of were aspergillosis (range: 2–28 days) [9,10].

Making the diagnosis of IPA can be challenging, especially among patients not considered classically at greatest risk (e.g., hematologic malignancy and stem cell transplant patients). Among the four European CAPA case series discussed, *Aspergillus* species were cultured in 78% of respiratory specimens; positive specimens included: 14 bronchoalveolar lavage, six tracheal aspirate and one sputum [[7], [8], [9], [10]]. Bronchoscopy in COVID-19 patients is often not performed due to the risk of aerosol generation. Further complicating IPA diagnosis, serum GM testing is often negative among COVID-19 patients, and sensitivity is low among ICU patients in general; in the published cases series, of the 22 cases tested, only three (14%) had positive serum GM tests [14,15]. Most COVID-19 patients lack known risk factors for IPA [4]. Thus, isolation of *Aspergillus* from a non-sterile site and negative serum GM should not rule out invasive disease. Clinicians may want to consider mold-active azole therapy at first indication of aspergillosis in COVID-19 patients. Our patient had a high serum GM index (>1.0), which is the recommended cutoff, and mycological criteria for probable IPA classification by the European Organization for Research and Treatment of Cancer and the Mycoses Study Group Education and Research Consortium [16].

Challenges in selecting a case definition for CAPA are also shared by the similar IAPA. For this reason, an expert panel recently proposed a new case definition for IAPA [17,18]. The definitions distinguish between invasive *Aspergillus* tracheobronchitis and other pulmonary manifestations and mainly rely on culture and GM to identify IPA. The expert panel stated that the IAPA case definition might also be useful to classify CAPA cases. Using this new definition, our case would be classified as probable CAPA [17]. A follow-up serum GM showed decreasing antigen titer which is consistent with antifungal treatment response.

Little is known about the pathogenesis of CAPA, and there is a need to characterize risk factors for the development of IPA. Although influenza infection was found to be an independent risk factor for IPA [4], the use of corticosteroids in patients with influenza is also a risk factor for IAPA [19]. A recent preliminary report suggested that dexamethasone was associated with reduced mortality in COVID-19 patients [20]. However, corticosteroids might be associated with adverse effects such as delayed viral clearance and secondary infections. Notably, of the four proven IPA cases reported during the previous severe acute respiratory syndrome (SARS) epidemic, all were associated with concomitant corticosteroid therapy [5,21].

In our COVID-19 case with probable CAPA, we observed co-infections with bacterial and additional fungal pathogens (*E. faecalis*, *A. baumanii,* coagulase-negative staphylococci, and *C. lusitaniae*), which complicated patient treatment. Previous reports of CAPA cases did not describe the presence of other microbial co-infections [[7], [8], [9], [10], [11], [12], [13]], although these are commonly encountered in patients with influenza. Previous reports of ICU patients with severe influenza frequently reported secondary bacterial infection, and these were significantly associated with IAPA in studies comparing severe influenza patients with and without IAPA (61% vs 31%; *p* < 0.01) [22]. Co-infections were reported in up to 50% of patients with severe coronavirus infections causing both SARS and Middle East respiratory syndrome (MERS) [23]. However, early data suggest that coinfection frequency might be lower in COVID-19 [[23], [24], [25]].

We present a case of COVID-19 associated pulmonary aspergillosis from Argentina, with a clinical course and attributes similar to previously reported cases, that highlights the challenges in diagnosing and treating these CAPA patients. The use of a clinical algorithm to discriminate *Aspergillus* respiratory tract colonization from invasive pulmonary aspergillosis in critically ill patients is warranted, such as the one developed for IAPA [17,18]. This is the first report of ventilator-associated pneumonia involving *A. flavus* in a patient with COVID-19 and ARDS in the Americas. Awareness of CAPA and accessibility to appropriate diagnostic tests are necessary for accurate and timely identification of this co-infection [26,27].

## Ethical considerations

Publication of this case report was approved by the Hospital de Clínicas “José de San Martin” IRB.

## Declaration of competing interest

All authors no reported conflicts of interest. The findings and conclusions in this report are those of the authors and do not necessarily represent the official position of the CDC.
